# Zooming of states and parameters using a lumping approach including back-translation

**DOI:** 10.1186/1752-0509-4-28

**Published:** 2010-03-18

**Authors:** Mikael Sunnåker, Henning Schmidt, Mats Jirstrand, Gunnar Cedersund

**Affiliations:** 1 Fraunhofer-Chalmers Research Centre for Industrial Mathematics, 412 88 Gothenburg, Sweden; 2 Department of Clinical and Experimental Medicine, Diabetes and Integrative Systems Biology, Linköping University, 581 85 Linköping, Sweden; 3 Freiburg Institute of Advanced Sciences, Freiburg University, D79104, Freiburg, Germany; 4 Department of Mathematical Sciences, Gothenburg University, 41296, Gothenburg, Sweden

## Abstract

**Background:**

Systems biology models tend to become large since biological systems often consist of complex networks of interacting components, and since the models usually are developed to reflect various mechanistic assumptions of those networks. Nevertheless, not all aspects of the model are equally interesting in a given setting, and normally there are parts that can be reduced without affecting the relevant model performance. There are many methods for model reduction, but few or none of them allow for a restoration of the details of the original model after the simplified model has been simulated.

**Results:**

We present a reduction method that allows for such a back-translation from the reduced to the original model. The method is based on lumping of states, and includes a general and formal algorithm for both determining appropriate lumps, and for calculating the analytical back-translation formulas. The lumping makes use of efficient methods from graph-theory and ϵ-decomposition and is derived and exemplified on two published models for fluorescence emission in photosynthesis. The bigger of these models is reduced from 26 to 6 states, with a negligible deviation from the reduced model simulations, both when comparing simulations in the states of the reduced model and when comparing back-translated simulations in the states of the original model. The method is developed in a linear setting, but we exemplify how the same concepts and approaches can be applied to non-linear problems. Importantly, the method automatically provides a reduced model with back-translations. Also, the method is implemented as a part of the systems biology toolbox for matlab, and the matlab scripts for the examples in this paper are available in the supplementary material.

**Conclusions:**

Our novel lumping methodology allows for both automatic reduction of states using lumping, and for analytical retrieval of the original states and parameters without performing a new simulation. The two models can thus be considered as two degrees of zooming of the same model. This is a conceptually new development of model reduction approaches, which we think will stimulate much further research and will prove to be very useful in future modelling projects.

## Background

### Model Reduction in Systems Biology

Systems biology is a rapidly growing discipline, which often is predicted to end up transforming biology into a field similar to physics and engineering - which integrates mathematical modelling in almost all their studies. Systems biology models are typically based on the mechanistic understanding of the systems. In other words, the model formalises a mechanistic hypothesis, which then is evaluated with respect to the available data. The potential of using a model for such an evaluation is that the evaluation can be made in a much more systematic and objective fashion, since complex interaction networks quickly become too complex to grasp using classical biochemical reasoning.

However, many relevant hypotheses are too complex to be analysed in a satisfactory manner, even when using modelling. Even with the power of super-computers, parameter and state spaces quickly become too large to be searched convincingly. Further, even though experimental data are being produced in ever larger amounts, most models are still highly over-parametrised with respect to the available data. This means that many or all conclusions regarding a model will often be highly uncertain, sometimes even arbitrarily uncertain[1]. Finally, even if a complex model could be validated from a computational point-of-view, an intuitive understanding of what actually goes on in the model is often easiest obtained from a simplified version of the model. All of these are reasons why model reduction is a central ingredient of systems biology modelling.

There are a number of existing approaches to model reduction. One important approach seeks to reduce the model as much as possible while preserving the input-output relations. An important example of this approach is balanced truncation, in which one derives transformed ordered states: the first transformed state is both the one affected the most by the inputs, and the state that affects the outputs the most. Therefore the states can be eliminated from the end with a minimal effect on the input-output relationship. This method is straightforward for linear systems [2], and different nonlinear extensions are available [3]. The main advantage of taking an input-output approach is that one optimally eliminates the unidentifiable and unobservable parts of the model. This means that much of the ambiguity regarding model predictions mentioned above is eliminated. However, the drawback is that the interpretation of the remaining states, i.e., those not considered as outputs, usually is lost. Since systems biology primarily uses modelling to evaluate mechanistic assumptions regarding the internal wiring of a system (unlike in many technical applications which primarily uses modelling to achieve a predictor) this is a major drawback. One way to partly circumvent this problem is to start by sub-dividing the model into modules, where only those modules are reduced whose interior details are not of interest to the particular situation [4,5].

Another approach focuses on the internal dynamics irrespectively of the input-outputs, and such methods are commonly applied to biochemical models. An important sub-class is based on time-scale separation. If the time-scales in a model are widely different, the model can usually be simplified so that all aspects that are not on the time-scale of interest are eliminated: slow processes are replaced by constants, and fast processes are assumed to be infinitely fast. The latter means that the fast processes are projected down to their corresponding steady-state behaviours, which can be expressed as analytical (or numerical) relations involving the other states. This is the basic principle behind computational singular perturbation and manifold reduction [6-9]. A problem is that the resulting model is usually not very much less complex in most aspects, since the fast variables still have to be calculated, and usually through complex (but algebraic) expressions.

Yet another approach based on time-scale separation, but which usually completely eliminates the non-dynamic states, is known as lumping [8,10]. Lumping is a widely adopted approach, since it can be done empirically [11], and it is also the central approach in this paper. Shortly, lumping is centred around the identification of pools of variables that can be approximated by a single lumped variable, where the internal distributions among the different sub-states are either irrelevant or assumed to occur momentaneously. Lumping may be either proper or improper [8]. Proper lumping refers to the case when the pools are non-overlapping. Here it is important to note that although most systems biology methods are proper, since improper lumps are rarely biochemically interpretable, there are important exceptions.

For instance, biochemically interpretable improper lumps appear in some reductions works dealing with the important problem of combinatorial complexity. In these works the lumps are not fully specified signalling intermediates, referred to as mesoscopic states [12-15]. In [15] these reductions were shown to be exact, i.e., they do not involve any approximations, and only make use of structural properties of the model [8]. In this paper we will deal with proper, in-exact lumping, which primarily is developed for linear (e.g., mono-molecular) systems. There are a number of related studies devoted to such lumping, e.g. [16-19]. However, other basic assumptions, or the basic algorithmic approach, are different compared to our setting, as e.g. [17], where a total separation of time-scales is assumed. Some more details regarding similarities and differences are pointed out as we derive our results below.

Another important sub-class of methods based on the dynamics are centred around a sensitivity analysis. Here the model components that affect the dynamics the least are eliminated. A model component could for instance consist of a state variable [20], an interaction [21], or a term in an expression [22,23]. Likewise, the considered dynamics can range from some overall qualitative property (like oscillations) [20], to the quantification of some specific outputs [8] (this latter option thus borders to the input-output approaches mentioned above). Sensitivity-based reductions are intuitive and easy to understand and implement, but they may have the problem of generating a model structure that cannot be interpreted biochemically [20]. Also, like most methods it does not find all kinds of reduction possibilities - for instance when two terms could be merged into one - and should therefore be combined with other approaches.

However, independently of which reduction approach one follows, there is one thing that would be very valuable, and which is rarely known in current versions of the methods: back-translation. Back-translation is a concept introduced in relation to core-box modelling [1,24,25], which is an approach that tries to combine the strengths of input-output approaches (reduced unidentifiability) with the strengths of dynamical reduction methods (preserved interpretability). A core-box model is the combination of a reduced core model, with known identifiable properties, and a mechanistically detailed model. The two models both describe the same system and experimental data. The combination of the models occurs through the back-translation, which is a mapping of the states and parameters in one of the models to the corresponding states and parameters in the other model. This mapping allows the two models to be considered as two versions of the same model, as two degrees of zooming. This property is of course extremely useful, since it allows the user to work with (e.g. simulate) the degree of zooming that is most appropriate for the current question, and then easily and quickly convert that model, or a part of the model, to another degree of zooming, if the question should change (for instance if one would suddenly be interested in the details of a specific sub-process). Nevertheless, there are very few available methods that derive such back-translation relations along with the reduction.

In this article we present a novel method for reduction that does provide such a back-translation. The method is based on lumping, and we present both the lumping step and the back-translation calculations. The method is derived in a linear setting, and is applied to two examples of previously published linear models for processes involved in photosynthesis. We also illustrate how the same concepts can be extended to a non-linear setting through the reduction of a system of differential equations for glucose transport. Finally, we discuss the strengths and short-comings of both the linear and the non-linear versions of our proposed methodology.

### Ordinary differential equations

We will study models described by ordinary differential equations (ODEs). Let the dynamic states be denoted **x**, the parameters **p**, the inputs **u**, and the outputs y, where **x **∈ *R*^*n *^and **y **∈ *R*^*l*^. Finally, let the the functional relations for the dynamics and the outputs be denoted **f **and **g**, respectively. Then, the set of ODEs is given by(1a)

Note that all these symbols are vectors, which is indicated by the symbols being bold. Note also that **x**, **u**, and **y **depend on the time, denoted by *t*, but that the explicit time-dependence is dropped from the notation except for in equation (1c). Note also that **x**_0 _may be parametrised and hence is a function of **p**. Equation (1) is referred to as a model structure, which is denoted ℳ.

For the theoretical developments, we will in this paper primarily focus on the special case of linear ODEs. Then the general nonlinear functional relationships **f **and **g **can be replaced by matrices **A, B, C, D **of appropriate dimensions as follows(2a)

### The usage of linear models in systems biology

The use of linear dynamic models in systems biology may at first sight look as an overly simplified approach. However, many biological and biochemical systems operate close to a steady-state where a linearised version of a detailed non-linear model well describes the dynamics of the deviations of the model variables around such a steady-state. How to linearise a nonlinear model around a steady-state (or trajectory) is described in many textbooks on basic systems theory, see for example [26-28]. The use of linearisation of nonlinear dynamic systems to obtain a linear model, with a limited range of validity, which facilitates theoretical analysis is a very common approach in engineering fields such as systems and control theory, electrical circuit theory (small signal analysis), rotating machinery and vibrational analysis. It should also be noted that the field of metabolic control analysis [29] is developed under the assumption that the underlying pathway or network is operating at steady-state.

The steady-state assumption is fulfilled in many metabolic pathways or networks at normal operation. This is because the fluxes through the network are often fairly constant, since several control mechanisms provide means to maintain a steady-state or homeostasis [29,30]. For oscillating systems it might also be possible to linearise submodels of the non-linear models [17]. Another important situation where linearity is an appropriate assumption is the case of probabilistic networks, where a state corresponds to the probability of a specific molecular conformation and a transition between states corresponds to a flux of probability [31]. This latter situation is the case in the prototype examples studied in this paper, where models for fluorescence emission in photosynthesis have been used.

There is also a relevant recent method for model reduction described in [32], which converts a non-linear system into an ensemble of (delayed) piecewise affine systems. Here the steady-state assumption is no longer critical, but the different (delayed) piecewise systems are valid during different time intervals. That model reduction process could in some cases be taken one step further by applying the lumping techniques described in this paper to each (delayed) piecewise affine system in the ensemble.

Finally, it should also be mentioned that there are several recently published papers on yeast osmo-regulation, where linear models and techniques have successfully been applied to the study of mechanistic details, even though the underlying system is operating far from equilibrium [33,34]. Note also the discussion in [17], concerning the possibility of applying the algorithms for linear (mono-molecular) models to pseudo-linear (pseudo-monomolecular) sub-models of nonlinear models, for which the internal reactions are functions of the external variables.

### Graph theory

The method developed below will make frequent use of concepts and results from graph theory. Since these might not be common knowledge for the average systems biology reader, we here give a short introduction. A graph consists of nodes, which usually are represented as dots, and edges, which connect some of the nodes. If the edges have a direction, we speak of a directed graph, or a digraph. The indegree and outdegree of a node in a digraph is the number of edges leading to and from the node, respectively. A directed graph is strongly connected if there is a path of edges leading from each node to each other node. A sub-graph is a sub-set of nodes and edges to the original graph, where only edges between nodes appearing in the original graph may appear in the sub-graph. If a directed graph is not strongly connected, there is always at least one sub-graph that is strongly connected (possibly consisting of a single node). Such a sub-graph that is as large as possible, a maximal strongly connected subdigraph, is referred to as a strong component (SC). Identification of SCs is a part of the method developed below. There are general and automatic methods for identification of SCs [35], but for the cases in this paper the SCs can be found by mere inspection of the graph. A node with a nonzero indegree, but a zero outdegree, is referred to as a sink (or target). A strong component from which there are no edges leading to nodes outside those of the strong component, but edges leading to the strong component, will here be referred to as a sink cluster. We will refer to a SC that is not a sink cluster as non-sink SC. Formally a graph *G *= (*N, E*) is a mathematical structure that consists of two sets *N *and *E*, where the elements of *N *and *E *are the nodes and edges of the graph, respectively, and E consists of 2-element subsets of *N*.

Let us now illustrate the graph-theoretical concepts above. In the directed graph *G*_1 _= (*N*_1_, *E*_1_),

there are five nodes and five edges, so that *N*_1 _= {*S*_1_, *S*_2_, *S*_3_, *S*_4_, *S*_5_} and *E*_1 _= {*a, b, c, d, e*}. The sub-graph ,

where  = {*S*_2_, *S*_3_, *S*_5_} and  = {*b*, *d*, *e*}, is strongly connected, since there is a path from each node to each other node. This graph is also a maximal strongly connected sub-graph, or a strong component, since any larger sub-graph would not be strongly connected. Node *S*_4 _has a zero outdegree and non-zero indegree, which makes it a sink. Now consider graph *G*_2 _= (*N*_2_, *E*_2_),

which has the same structure as graph *G*_1 _apart from the addition of an edge f, which leads from node *S*_4 _to node *S*_3_. The sub-graph ,

where  = {*S*_2_, *S*_3_, *S*_5_} and  = {*b*, *d*, *e*}, is strongly connected but not a strong component. This is because a larger sub-graph in which node *S*_4 _and edges c and f are included is also strongly connected. Note that node *S*_4 _in *G*_2 _has a non-zero outdegree and is for this reason not a sink. The sub-graph ,

where  = {*S*_2_, *S*_3_, *S*_4_, *S*_5_} and  = {*b*, *c*, *d*, *e*, *f*}, is a strong component. Since there are no edges leading from the strong component to other nodes (*S*_1_), but only in the opposite direction,  also constitutes a sink cluster as defined above.

In this paper, graphs are used for illustration of biochemical systems, where the nodes represent the involved biochemical entities and the edges represent transformations between these entities such as reactions, translocations, or conformational changes.

## Methods

### Initial Observations

We will in this section make some initial observations, which form the conceptual basis for the model reduction approach presented in this paper.

Consider two linear model structures ℳ_*g *_and ℳ_*c *_where one of the states in ℳ_*c *_corresponds to a group of states in ℳ_*g*_. We can think of ℳ_*c *_as a reduction of ℳ_*g *_in which the states of the pool have been lumped.

For example let the graph *G*_1 _above represent a model structure . If the states corresponding to *S*_2_, *S*_3 _and *S*_5 _are lumped we get the reduced model structure , which can be represented by

Where  and  have the same interpretation as *S*_1 _and *S*_4 _in *G*_1_, respectively, and where  corresponds to the lumped state.

In general, let the states in the pool in ℳ_*g *_be denoted *x*_1 _to *x*_*n *_and let the corresponding lumped state in ℳ_*c *_be denoted . Let the reaction rate from a state *x*_*q *_to a state *x*_*p *_be denoted *v*_*pq *_so that(3)

Where *k*_*pq *_is a kinetic parameter. In ℳ_*c *_the reaction rate from  to a state external to the pool *x*_*j *_is denoted  and is given by(4)

Reaction rate  corresponds to the sum of all reaction rates in ℳ_*g *_from any of the pooled states to state *x*_*j*_, which is denoted *v*_*j*_, and is given by(5)

Note that if there is no reaction between some state *x*_*i *_in the pool and the state *x*_*j*_, *k*_*ji *_is equal to zero. Since *v*_*j *_and  have the same interpretation in the two model structures, we can set them equal and solve for one of the parameters, e.g., (6)

Now, note that Eq. (6) gives a translation between parameters of the original and the reduced model structure. The translation requires the fraction of each state variable of the pool, *x*_*i*_, to the lumped state variable, , to be known. Such fractions are therefore useful auxiliaries to derive for reduction of models by lumping. Define the fraction parameters *η*_*i *_as(7)

so that Eq. (6) takes the form(8)

Note that in general the fraction parameters are time-varying. However, internal equilibrium can be assumed for a sub-system of states with fast reactions. This is justified by terms on the right hand side of Eq. (2a) that are large enough to neglect the derivative term on the left hand side. The sub-system is then said to be in quasi-steady-state (QSS). Variables in such a sub-system will show a high correlation in QSS, leading to approximately time-invariant fraction parameters. By definition from Eq. (7) the states in ℳ_*g *_that are lumped have a clear interpretation in the reduced model structure, ℳ_*c*_, given by(9)

i.e., each of the lumped variables corresponds to a given fraction of the lump .

In summary, the basic observation is that the relation between the states in the original and the reduced model is constant over time, and is given analytically by Eq. (9). This is possible because of the basic assumption behind lumping: that the states within the lump reaches internal equilibrium momentaneously.

**Example 1**. *To illustrate the ideas above, let us consider the six-states model depicted below*.

*This model is used to predict the observed behaviour of fluorescence emission in photosynthesis *[31], *which is characterised by a flux of probability between the states. To translate the figure above to a model structure in the form of (2), all rates are assumed to be given by expressions proportional to the probabilities of the states they emanate from, which would correspond to mass action kinetics for a regular biochemical reaction network. This makes the resulting model structure linear. Let the model structure be denoted , with states x*_1_*to x*_6_, *where the initial condition for state x*_2_*is set to *1 *and for the other states to *0. *The values of the kinetic parameters in *[31]*are k*_12 _= 6.72, *k*_13 _= 6.72, *k*_32 _= 1920, *k*_23 _= 2500, *k*_43 _= 50000, *k*_34 _= 50000, *k*_54 _= 25000, *k*_45 _= 6700, *k*_65 _= 480 *and k*_56 _= 240. *Since the order of magnitude of the kinetic parameters are k*_32_, *k*_23_, *k*_43_, *k*_34_, *k*_54_, *k*_45_, *k*_65_, *k*_32 _>> 1 s^-1 ^*and k*_12_, *k*_13 _≈ 1 s^-1^, *the reactions between states x*_2 _*to x*_6 _*are all reversible and fast compared to a time-scale of seconds, whereas the reactions to state x*_1 _*operate on this time scale. The equation for the output, y, is*(10)

*By lumping states x*_2 _*to x*_6 _*in **into one state *(11)

*we obtain the reduced model structure  as shown below*.

*Note that **and that **corresponds to the sum of states x*_2 _*to x*_6_. *All probability is initially assigned to state x*_2_, *which gives the following equations for the reduced model*.(12)

*The translation between the reduced and original parameters are given by Eq*. (8)(15)

*Where **and **so that **and *. *However, we still lack analytical formulas for the fraction parameters η (which can be expressed as a function of the parameters alone). We also lack an automatic method for conducting the model reduction. These two things will be derived in the following sections*.

### Lumping of States

We will now generalise the above observations to a concrete methodology, with the goal that it should be useful in practice. In particular, the method should

A Automatically identify lumps in a model

B Deliver analytical formulas for back-translation

In this section we focus on step A, which we will sub-divide in several sub-steps, and in the next section we turn to the calculation of back-translation formulas.

A basic criteria for lumping a group of states is that the reactions between states in the group occur on a much faster time-scale than that of interest to the modeller. A group of states that are (weakly) connected by such fast reactions will here be referred to as a *directed graph of fast reactions *(DGFR). To identify the non-connected DGFRs of a linear model structure, ℳ_*g*_, we first rewrite Eq. (2a) on the form(16)

where the kinetic parameters of **p**_*f *_are those of **p **that are much larger than the inverse of the time-scale of interest, and *dim*(**p**_*f*_) + *dim*(**p**_*s*_) = *dim*(**p**), where *dim*(**z**) gives the dimension of a vector **z**. The method of ϵ-decomposition [36] can then be applied by permuting **A**_*f *_into block diagonal form. The blocks of non-zero elements can be interpreted as corresponding to the various non-connected DGFRs of the model. For example, if the reactions corresponding to edges b, d, and e in graph *G*_1 _above are large and the other reactions slow, the matrix **A**_*f *_for the corresponding model structure can be permuted into the block diagonal form. This is done by exchanging the rows and columns for the states corresponding to *S*_4 _and *S*_5_, so that the permuted matrix  takes the form(17)

so we have identified one DGFR consisting of three states and two states (corresponding to *S*_1 _and *S*_4_) that are not connected to other states by fast reactions. Note that some of the block elements in Eq. (17) are equal to zero if the reactions between the corresponding states are missing, and that Eq. (17) is only used to illustrate the matrix form in principle.

To find a permutation for the purpose of identifying DGFRs we can construct a matrix **M **in which each element, *M*_*ij*_, is assigned a value 1 if *A*_*fij *_or *A*_*fji *_are non-zero and 0 otherwise. The total connectivity

matrix **M**_*c*_(18)

then reveals if there is a path between each two states. Note that equation (18) contains both multiplications of the matrix **M **with itself, and summations of such products. The states *x*_*i *_and *x*_*j *_are connected if and only if *M*_*cij *_≠ 0. Using the connectivity matrix it is thus straightforward to determine the permutation and the independent blocks of .

These independent blocks, or DGFRs, determined by the ϵ-decomposition must be analysed further, before useful lumps can be identified. The reason for this is illustrated by the following digraph

where *k*_*s *_≈ 1*s*^-1 ^and *k*_*f *_≫ 1*s*^-1^. This digraph could have been generated as a DGFR from an is ϵ-decomposition, even though *k*_*s *_is slow. The reason for this is that there is a fast reaction from state *x*_2 _to *x*_1_. The consequence of this slow reaction in the DGFR is that we cannot assume QSS for this DGFR directly, since this would imply that inputs to states *x*_1 _and *x*_2 _would instantly end up in *x*_3 _(since *x*_3 _is a sink).

This further analysis of the DGFRs starts with the characterisation of strong components (SCs) in each DGFR. Recall that an SC is a maximal strongly connected sub-digraph in which each node can be reached from each other node. Note that there is at least one SC (possibly including only one or all states) that acts as a sink cluster, meaning that the corresponding states will be assigned a non-zero value in QSS. Note that if there is a path of fast reactions from a non-sink SC to a sink cluster, but no path in the opposite direction, the states of the non-sink SC will have little effect on the overall model dynamics. Unless the modeller has a particular interest in these non-sink SC states, e.g., if they are important for the observation function, the non-sink SC and the sink cluster can be lumped. The states of the non-sink SC will then be assigned a zero value at all times in the back-translation.

It is important that there is only one sink cluster within each pool of states that are lumped, since the dynamics on the time-scale of interest might otherwise affect the fraction parameters. If there is a path of fast reactions from a non-sink SC to more than one sink cluster, the rule of thumb is to avoid lumping the non-sink SC to any of the sink clusters. However, if there is a path of slow reactions from only one of the sink clusters to the non-sink SC, the corresponding states can be lumped. Finally, we also identify which states are slow, i.e., can be treated as constant at the time scale of interest. A simple criterion for identification of such states in linear models is that a state *x*_*i *_is slow if max(*k*_*ji*_) << ϵ ∀ *j *and max(*k*_*ji*_) << ϵ ∀ *j*. This statement can formally be connected to the eigenvalues of the system matrix through the Gershgorin circle theorem, see for example [37]. If there is a union of *k *Gershgorin discs corresponding to "small" diagonal elements with "small" row and column elements and this union is disjoint from the other Gershgorin discs, the corresponding states are slow. Alternatively, we could also check if the state remains approximately constant at a given time scale, in a simulation of the original model. Note that in the case of oscillating systems, a more suitable method is the method of averaging [17]. Below we summarise the most important steps in the identification of a states lumping scheme.

A1 Use ϵ-decomposition to identify non-connected digraphs of fast reactions (DGFRs).

A2 Identify the strong components (SCs) in each of the DGFRs.

A3 Classify the SCs as sink clusters or non-sink SCs.

A4 Check if the non-sink SCs can be lumped with any of the sink clusters.

A5 Identify slow states in the reduced model.

These steps make up a lumping scheme for the state variables.

**Example 2**. *For the six-states photosynthesis model, ϵ-decomposition gives one DGFR consisting of states x*_2 _*to x*_6_*(step A1). These states are all interconnected with fast reactions, and thus constitute a SC (step A2). Since there are no fast reactions to states outside the SC, it is classified as a sink cluster (step A3). There are no non-sink SCs in the DGFR and we do not need to consider the additional lumping of non-sink SCs (step A4), and there are no slow states in the reduced model (step A5). Having followed the steps 1-4 above, we have thus identified a suitable lump consisting of states x*_2_*to x*_6_, *which in the lumped model will correspond to the state *.

### Fraction Parameters

We now turn to the problem B above: deriving back-translation formulas. This means that we are seeking analytical expressions for the fraction parameters *η *in Eqs. (8) and (9). The derivation presented here relies on that QSS can be assumed for each group of lumped states, which is ensured by the work flow in the previous section.

Consider a sub-model of states to be lumped, with the corresponding system of differential equations(19)

where *dim*(**x**_*l*_) = *m *> 1 and **u**_*l *_are the inputs to the lumped states. Recall that **u**_*l *_are slow compared to the dynamics within the lump, since we assume QSS. Using the notation *qss *for quasi-steady-state, this assumption gives(21)

so that(22)

where *η*_*li *_is the fraction parameter for the i:th lumped state variable.

Eq. (21) requires **A**_*l *_to be invertible, which is not always the case. **A**_*l *_is not invertible in the absence of output reactions, i.e., with no reactions *v*_*ij*_, where *x*_*j *_is in the lump but *x*_*i *_is not. The reason for this is that the dynamics given by **A**_*l *_then introduces an apparent conserved moiety: that all the states in the lump sum up to . This is a true conserved moiety if there are also no inputs, since then  is constant over time, and the only thing that can happen within the lump is re-distribution. With such a conserved moiety one of the rows of **A **can be expressed as a linear combination of the others, i.e., **A**_*l *_is not invertible. It should be noted that also in the situation of zero inputs **u **but with outputs, the matrix **A**_*l *_will have the same problem (since the inputs are filtered through the matrix *B*), and thus non-invertible.

Fortunately, also in the situation of non-invertible **A**, our method can be applied. Since the non-invertibility is due to the presence of a conserved moiety, there is an additional relation that can be used: the one describing the moiety.(23)

Note that there can only be one conserved moiety in a given connected linear system. Equation (23) can be used to replace one of the rows in the matrix equation (21). Replacing the last row in Eq. (21) with Eq. (23) gives(24)

Where(25)

The fraction parameters can in this case be obtained by substitution of  from Eq. (24) into Eq. (22).

Note that if Eqs. (24)-(27) are used,  is a function of the lumped state variable . However,  appears linearly in each entry of  and therefore disappears in the division in Eq. (22). So the fraction parameters are independent of , which is typically varying over time. Hence the fraction parameters are time-invariant, just as we want. This should be compared with Eq. 22 och Eq. 23 in [17] and the recipe proposed by Clarke [38].

The quasi-steady-state equation, Eq. (24), can be solved explicitly since the solution only requires the inverse of the non-singular matrix  and the multiplication with the matrix  and a vector . Note that these matrices and vectors are functions independent of the model states. The use of symbolic tools are advantageous, since analytical formulas of the fraction parameters can be obtained. The inverse of a matrix can be computed with a number of symbolic software packages, such as Maple (also used by the Symbolic Math Toolbox for Matlab until version 2009a) or Mathematica. We have used the symbolic toolbox for Matlab in our examples with a good result for all matrices tested. However, since fraction parameters are time invariant for linear models, if we are only interested in the numerical values, we can also substitute the values of the parameters in the matrix . Matlab provides very efficient algorithms for matrix inversion of numerical matrices, based on the Fortran package LAPACK. This makes the algorithm much faster than for symbolical solution of the QSS equations.

The complexity of solving the QSS equations boils down to the problem of symbolical inversion of a matrix. For all our test cases the time for symbolical matrix inversion was rather short (the total model reduction program time was less than 45 s on a 2.19 GHz PC). In [17] the assumption of a multi-scale nature of the model was exploited to reduce the number of parameters. A similar approach may be used for some of the parameters also without the strong assumption that all parameter values are clearly separated. Also, in [17] it was suggested to choose the dominant terms in the solutions as functions (monomials) of the parameters, which again relies on separated time scales in the model.

**Example 3**.

*To illustrate the ideas of this section we again consider the six states model for fluorescence emission inphotosynthesis. We have already identified states x*_2_-*x*_6_*as a suitable states to be lumped. Since there are no inputs to states x*_2 _*to x*_6_, *Eqs*. (24)-(27)*are used to compute the fraction parameters. The matrix  takes the form*(28)

Note that we have approximated(29)

*This is justified since these two parameters are much smaller than the others, and is done because it gives much simpler expressions for the fraction parameters derived below. The assumption (29) is however not necessary, and is not a part of the method per se, and it could be omitted with the only change that the approximation would be a little better, and that the expressions would be more complicated*. *and **are given by Eqs*. (26) *and *(27) *as*(30)

*The vector **is obtained from Eq*. (24) *and the fraction parameters from Eq. 22, so that*(32)

*The analytical expressions for the fraction parameters are determined by analytical inversion of ***A**, *which gives*(33)

Where(34)

**Figure 1:**
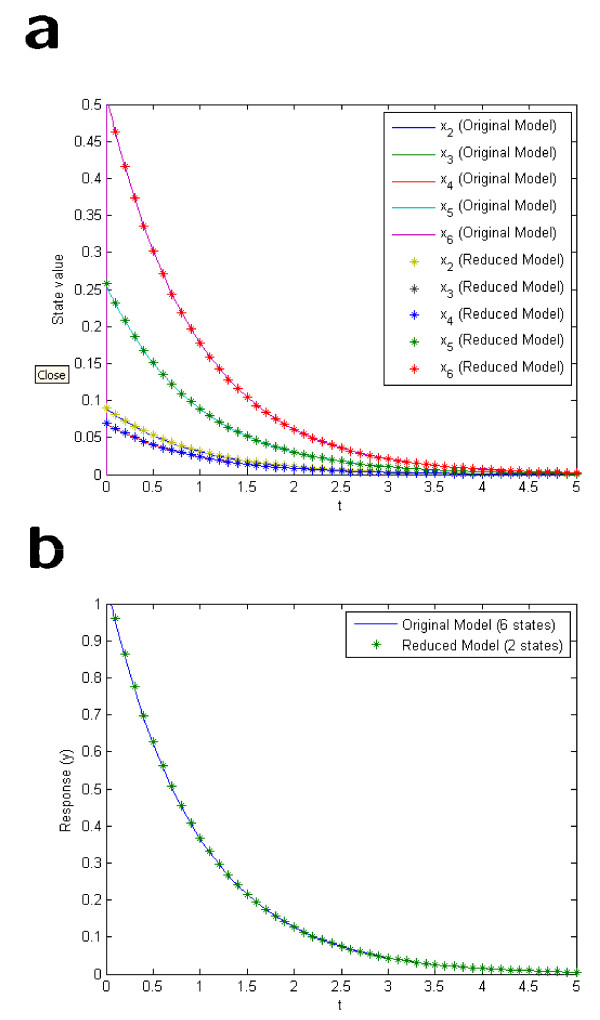
**Comparison of back-translated state trajectories from the reduced model and the corresponding output function to those of the original model**.

*Note that approximate trajectories for the states of **can be obtained from Eqs*. (9), (23) *and *(33), *if the solution to the model equations of **is available. The state trajectories resulting from back-translation and the output function are compared to those obtained by simulation of **in *Figure 1(a)*and *1(b), *respectively. As can be seen the disagreement is negligible at the time-scale of interest. This high agreement validates the assumption (29), but, above all, it validates our reduction method*.

### Implementation

The method has been implemented in matlab and is based on the systems biology toolbox, which is a freely available and widely used matlab toolbox (http://sbtoolbox.org, [39]). The Additional files 1, 2, 3 and 4 contain scripts for all the examples in this paper. The provided implementation is script-based, user-friendly and easily modified, and provides automatically reduced models from any correctly entered model to which the theory applies. The functionality will be included in the toolbox once the paper has been published.

## Results

### The Model

**Figure 2:**
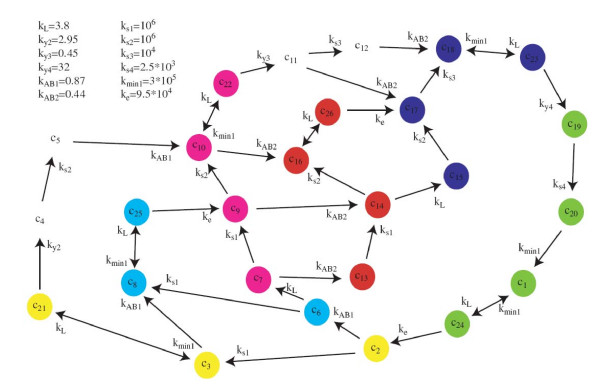
**The original 26-state model for photosynthesis used in the example**. The colouring corresponds to the lumps, such that lumps *L*_1_, to *L*_6_, corresponds to states coloured green, orange, blue, purple, red, and yellow, respectively. The four states without circles are not part of any lumps, but are eliminated from the reduced model.

We will now demonstrate the performance of the method based on lumping on a larger model for fluorescence emission in photosynthesis. This will also exemplify the shortcomings of a purely input-output based reduction method like balanced truncation in a systems biology context. The chosen model was originally published in [31,40], and it is based on mechanistic understanding of the corresponding biochemical system. The original model consists of the 52 states, but they are divided in two identical disjoint groups, and hence only one of these groups needs to be considered. The studied system therefore consists of 26 state variables, corresponding to 26 different configuration states of a protein specific complex. The state variable *x*_*i *_represents the probability that the protein complex is in the i:th configuration state (see Figure 2). At time zero all the probability is assigned to state *x*_1_, corresponding to the state of the molecule right after the excitement with a laser flash. Further, the input only enters in one of the state equations, only a single linear combination of the states may be measured, and the measurements are not directly affected by the input. More specifically Eqs. (2a)-(2c) take the form(35a)

Here **A **is a 26-by-26 dimensional matrix, and the *A*_*i, j *_element is given in Figure 2. This means that for instance the *A*_3,21 _element is given by *k*_*min*1_, that the *A*_4,21 _element is given by *k*_*y*2_, and that the *A*_3,4 _element is zero.

### Results with Balanced Truncation

**Figure 3:**
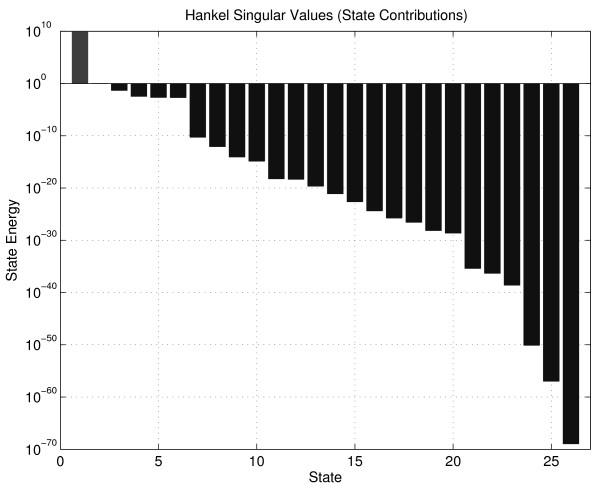
**Hankel singular values for the 26-states photosynthesis model**. Note that the first value is unstable.

**Figure 4:**
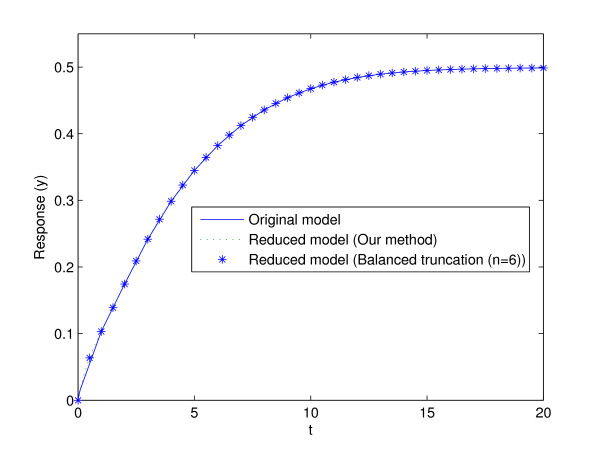
**Output from the original model and the two reduced models of 6:th order, obtained by the two reduction techniques**.

Balancing of (35) using the function hsvd in MATLAB gives the Hankel singular values depicted in Figure 3. The initial condition for state *x*_1 _was replaced by a Dirac delta function, as an input on the right hand side of the differential equation for this state. This was done to justify the input-output perspective of the method since there are no inputs to the system. As can be seen there is a jump after the first two states, another jump after 6 states, and after 10 states the singular values are below computer precision. That indicates that a 2-state model works reasonably well, that a 6-state model works good, and that there is no point in adding more states after the 10:th state has been added. This prediction is verified by the corresponding truncations, and by simulations comparing the original and the reduced model output (see Figure 4).

### Results with Lumping

Now our novel lumping approach is applied to the same model. The first step (step A1) is to identify the DGFRs of the model using 3-decomposition. Rate parameters much larger than 1000 *s*^-1 ^are considered to be fast, which is reasonable if we are interested in a time-scale of seconds. This gives five disjoint groups of states

However, the only external reactions to the states in  and  are from states *x*_21 _and *x*_22_, respectively. These state variables *x*_21 _and *x*_22 _have low values (smaller than 5 × 10^-6^, to be compared with the largest states with values that are close to 1) at all times, so the states in  and  are even smaller and can be removed from the model without affecting the overall model dynamics much.

The second step (step A2) is to identify the SCs in the DGFRs and in the third step (step A3) we check which of them are sink clusters. It turns out that there are no SCs in the model consisting of more than one state, but several sinks. The single state sinks in the three disjoint groups are states *x*_1 _and *x*_3 _in , states *x*_8_, and *x*_10 _in , and states *x*_16 _and *x*_18 _in . To conclude the lumping, the fourth step (step A4) checks which non-sink states that can be lumped with the sinks in their DGFR. We are not particularly interested in any of the states for which there is a path of fast reactions to a sink, but no path of slow reactions in the opposite direction. These states are therefore lumped with the corresponding sink state, as described in the guidelines for this step. There is an ambiguity for state *x*_24_, since there is a path of fast reactions from this state to two separate sinks, *x*_1 _and *x*_3_. However, there is a path of slow reactions from *x*_1 _to *x*_24_, but no path from *x*_3 _to *x*_24_, so *x*_24 _is lumped with *x*_1_. There are also paths of fast reactions from state *x*_25 _to the two states *x*_8 _and *x*_10_. With the same reasoning as for *x*_24 _above, we decide to let *x*_25 _be lumped with *x*_8_.

The procedure above gives rise to the following six groups of state variables(36)

and lumping the states of each group then results in a reduced model structure represented by the following digraph.(42)

Note that there are no slow states in the reduced model (step A5). We then calculate the back-translation formulas (Step B) using Eqs. (24)-(27).

**Figure 5:**
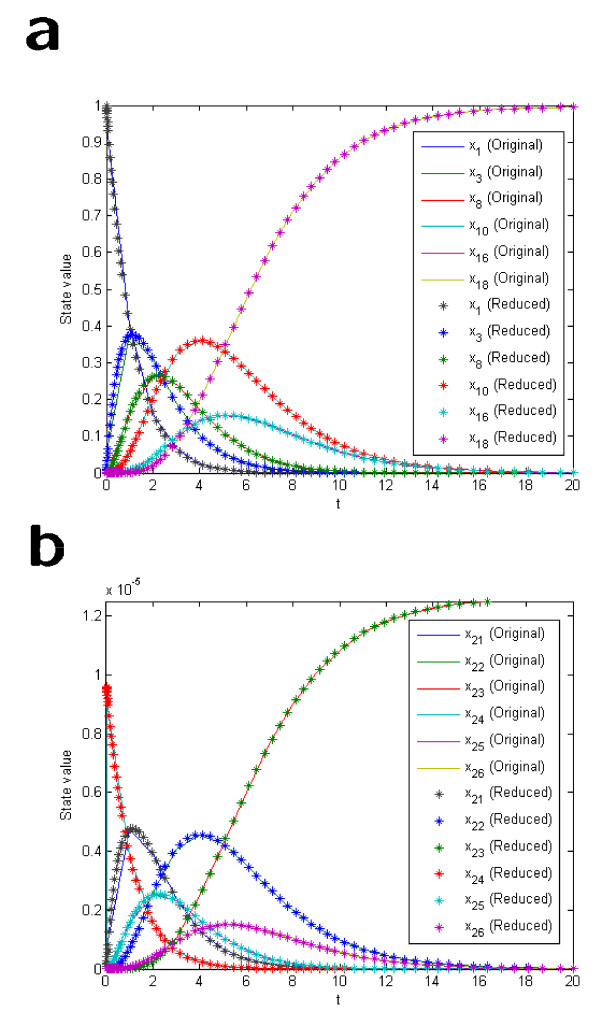
**Comparison with back-translated state trajectories from the lumped model with those of the original model**.

In Figure 4 the outputs from the original model and the reduced model are compared, and as can be seen the agreement is very good. Finally, Eqs. (9), (21), (22), and (36)-(41) can be used to compare the original states obtained with back-translation using fraction parameters from the reduced model with those of the original model. The results for the six states that are sinks, as well as the six states that are connected to them via reversible reactions, are shown in Figure 5(a) and 5(b), respectively. As can be seen the agreement is convincing.

## Nonlinear Model Reduction with Lumping

The aim of this section is to give some insights about the applicability of our method to nonlinear models.

### Reduction of a Model for Glucose Transport in Yeast

**Figure 6:**
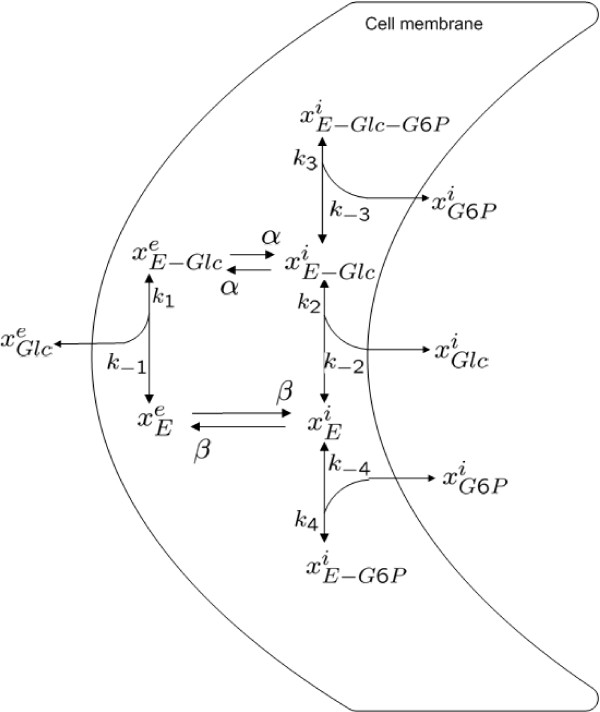
**Model for glucose transport in the yeast Saccharomyces cerevisiae**.

A model for glucose transport in the yeast *Saccharomyces cerevisiae *was presented in [41] and is depicted in Figure 6. The paper [41] presents this detailed state-space model with a number of assumptions, and reduces the complete system into a single rate-expression. We will now illustrate how one can use nonlinear analogues to the methodological steps introduced in this paper to derive fraction parameters, *η*, which again gives back-translation formulas; interestingly, the fraction parameters may also be used to easily calculate the in [41] obtained rate expression. The differential equations for the original model are given by(42a)

where it should be noted that the right hand side of the ODEs are nonlinear functions of the states. Recall that step A in our method is to identify a suitable lump. For this example, the reduction problem is stated such that the resulting reduced model should go from extracellular glucose to intracellular glucose. This means that all states in between should be lumped; this gives(43)

Note that this implies that the lump is given by all states including the carrier protein, and thus a conserved moiety.

Recall that step B in our method is to identify the fraction parameters for this lump. Also recall that this is done by identifying a linear matrix equation for the states in the lump, which can be inverted and used to calculate the steady-state values of the lumped states, which in turn gives the fraction parameters *η*. Finally, recall that this invertible matrix in the most general form is given by a combination of steady-state assumptions and the sum containing the conserved moiety.

In this nonlinear example, there are four reversible reactions in the model for the transport processes through the inner and outer parts of the cell membrane (Eqs. (42a)-(42d)). These were identified as fast in [41], i.e., the corresponding states are in quasi steady-state. A method for automatic detection of fast states, for which the quasi-steady-state assumption is fulfilled, was proposed in [17]. This means that the right hand side of the equations can be set equal to zero (QSS). We also assume that the total flux of the carrier molecules between the inner and outer walls of the cellular membrane is zero(44)

These assumptions put together gives us a nonlinear version of the matrix equation. More explicitly, the equations stating the QSS assumptions corresponding to Eqs. (42a)-(42d) followed by Eqs. (44) and (43) collected in a matrix gives(45)

where  and . Note that Eq. (45) has the same form as Eq. (24), but with variables as matrix elements. However, the variables in the matrix are all external to the lump, which means that these variables can be treated as parameters in the derivation of the fraction parameters and that Eq. (45) can be solved explicitly. This is a typical situation for nonlinear models based on mass-action kinetics, since the conserved moiety is usually only represented by one of the variables within each term of the polynomials. Symbolically inverting the matrix in Eq. (45) gives(46)

where(47)

Note again that this results in a relation between the individual states of the lump (those with the carrier E) and the total lump through fraction parameters, *η*.

Interestingly, these fraction parameters can be used to derive the in [41] obtained rate expression for the glucose flux into, and out of, the cell. Note that the only path for glucose to be transported between the outer and inner parts of the cell membrane is the rate-limiting reversible reaction between states  and . Note also that the rate of the reaction in either direction is proportional to the magnitude of the substrate state.

Now consider the reaction from  to . Since the carrier within the cell membrane is conserved at all times, the potential largest possible reaction rate, denoted *r*_*max*_, is obtained if all carrier molecules is in the state from which the reaction leads. However, typically only a fraction, , of the carrier molecules is in state  at a given time. Hence the rate of the reaction into the cell is . Similarly, we have for the flux out of the cell (efflux): . From Eqs. (46) and (47) we can thus derive the influx, *r*_*in*_, as(48)

and the efflux, *r*_*eff*_, as(49)

This flux rate expressions are the same as presented in [41]. This thus demonstrates how the same concepts we have derived for the linear case, can be applied also to calculations of fraction parameters for nonlinear systems, and how these fraction parameters can be used to solve other nonlinear problems.

## Discussion

In this paper we have presented a novel lumping approach. Apart from identifying suitable lumps in a novel and automatic way, the method also finds analytical expressions for the back-translation relations regarding both states and parameters between the original and the reduced models, resulting in the derivation of a zoomable model. The method has been applied to two models for fluorescence emission in photosynthesis, and we have demonstrated how the concepts supporting the method may be used also to nonlinear problems.

In the larger photosynthesis related model, a reduction was also performed using balanced truncation; let us now compare the performance of these two methods. A first observation is that the methods perform comparably when it comes to preservation of the input-output relations of the model. Both methods predict and supply good agreement between a reduced 6-state model and the original model of 26 states. Balanced truncation do provide reasonable model agreements also for lower order models, but it is not until 6 states are added that the agreement is equally good to the lumped 6-state model. However, even though the two methods yield comparable results from an input-output perspective, the lumping method has at least two important advantages when it comes to other properties of importance in a systems biology analysis.

First, the reduced model structure may be interpreted biochemically. As can be seen in Figure 2, the lumped variables correspond to combinations of neighbouring states. The analysis thus means that these neighbouring states may be treated as a single variable. Similarly, the reduced model structure above reveals that the actual dynamics of the original model can be predicted by a simple cyclic structure of six states. Finally, this also means that all future results and analyses that will be produced for the reduced model will have a more direct and clear interpretation.

The second reason why the proposed lumping approach is advantageous is that it provides an analytical back-translation formula for the mapping of the states and parameters in the reduced model to those of the original model. That means that one may not only interpret the results for the reduced model, but actually see what they mean for individual time-series and parameters in the original model. Such a back-translation may actually be said to result in a 'zoomable' model, which could for instance be used as a module in a larger object oriented model [42,43]. A zoomable model could for example be used in a larger model of the plant where photosynthesis is a sub-system. Such a larger object oriented model would probably be easiest to construct by combining reduced ('zoomed-out') versions of the models for the different sub-modules. If then a later analysis would require more details regarding the photosynthesis, one could simply switch to the 'zoomed-in' version of some part of the model, and examine the corresponding results - without performing a new simulation. This is an important advantage compared to conventional constructions of model libraries, typically used in object-oriented modelling [42]. To sum up the comparison between our new lumping approach and balanced truncation: the two methods perform similarly from an input-output perspective, but our novel method is much more useful in a systems biology context, since then an increased interpretability, through the lumping and the back-translation formulas, is highly advantageous.

A final note on the back-translation. The calculations for back-translation are relatively straight-forward, given a reduced model, and have been touched upon elsewhere [17]. However, the main point we want to make concerning the back-translation is a conceptual one; information about the original system can be retained from the reduced one without doing any new simulations. Also, the state-space trajectories from simulations between the original and back-translated states are compared. We are not aware of any similar previous comparisons for lumping-based methods in systems biology. However, back-translation-like formulas are at the heart of various time-scale based methods (apart from lumping), where the eliminated states are simply replaced by algebraic expressions. We also want to emphasize that both the reduction and the back-translation increases the interpretability and biochemical understanding of the model. Consider for instance our 26-state example, which has been reduced to a 6 dimensional model. These 6 states corresponds to disjoint groups of states in the original model, since our lumping is proper. Also, the relation between these groups is much more straightforward (a circular structure), compared to the original model, and time-traces for the original model can be retained without doing a new simulation. Nevertheless, some of the expressions might become complex and are not always themselves straightforward to interpret.

Our method can also produce multi-scale approximations of a linear model; just redo the model reduction for each specific value of 3 (in the 3-decomposition) corresponding to the time scales under study. This will generate a set of reduced models, which are valid at different time scales, and altogether form a multi-scale approximation of the original model. For previous work on multi-scale approximation of linear models, see [17]. However, for each time scale our method is guaranteed to produce a biologically interpretable model, without the strong assumption of clearly separable time scales for all parameters.

One shortcoming of the current version of the method is that states that are not part of a sink, and to which there is no path from a sink, do not have a proper back-translation. The reason for this is that these states give an insignificantly small impact on the model dynamics; their main purpose in the model are to serve as a short-lived intermediates. In other words, the QSS values, upon which the back-translation formulas are based, is zero. Nevertheless, if for some reason we are particularly interested in any such state, the state can be kept in the model simply by not lumping it with any of the other states; the price is one additional states in the reduced model. It should also be noted that our method deals with the inversion of a matrix, which might be a difficult task if the number of states is large. For larger models one might consider methods based on methods that only makes use of the order of magnitude of the parameters, such as that in [17]. Another shortcoming is that the back-translations do not yet allow for back-translation of uncertainties, e.g., obtained from parameter estimation given experimental data. This will have to be improved before the method can be fully useful in a core-box modelling situation [1]. Model reduction methods based on solving the quasi-steady-state equations usually generate models that are less stiff. However, the reduction in simulation time, for the reduced in comparison to the original model, was not large (< 5% for the models tested). One reason for this is the availability of good stiff ODE solvers, e.g., the Matlab solver ODE15s which was used in this paper. The main advantage of our method is instead that biologically interpretable reduced models, based on proper lumping, are guaranteed. Note that this is done without any strong assumption about the kinetic constants, which may be compared to the approach in [17]. The reduced model can always be represented graphically as a network of states, and lumped variables, where each state of the original model has a clear interpretation as a fraction of one lumped state (or possibly left unchanged). This makes it easy to discuss the reduced models with scientists who lack a strong mathematical background, which is very important for the success of large interdisciplinary projects that are typical in systems biology. It also gives the researcher a great overview of the individual parts and of the important interactions in the reduced model, which are responsible for the observed dynamics.

The method is developed in a linear setting, even though we have demonstrated how the main concepts and ideas may be extended to a non-linear setting. In the chosen nonlinear example, the lump was identified by the problem, and consisted of a conserved moiety. The moiety relation together with the usual assumptions of QSS within the lump allowed us to construct a matrix equation (45), which is analogous to the linear formula (24). Further, just as for the linear case, the matrix equation could be solved analytically using conventional computer algebra software, and with the resulting steady-state values identified, the fraction parameters can be calculated using the linear formula. All these central steps are thus more or less directly transferable between the linear and nonlinear situations. Interestingly, we also showed how these fraction parameters could be used to easily derive a complex Michaelis-Menten expression for the given system.

However, it should be noted that the extension to the nonlinear situation is not done without the introduction of new problems. Note for instance that the fraction parameters in this nonlinear model are functions of states variables and may therefore vary over time. Further, the states outside the lump in the reduced model can not be simulated after substitution of the fraction parameters into the model ODEs. The reason is that the right hand side of the ODEs is algebraically equal to zero, which gives the false impression that the outside system is always in steady-state. We are currently working on methods to resolve this, seemingly general problem in the reduction of nonlinear models, with promising results. All in all, we believe that the main contribution of this paper is the introduction of the concept of back-translation, and the demonstration that it is possible to derive back-translation formulas in an automatic manner. Since this is a fundamentally and conceptually novel improvement of lumping and model reduction, which is very useful in a systems biology context, we think that our contribution could stimulate much future research to improve our present contributions further.

## Conclusions

We have in this paper introduced a novel reduction method, which is centred around two main steps. The first step is an automatic identification of states that are suitable for lumping. These states are identified in four sub-steps, which makes use of existing and powerful concepts from graph-theory and ϵ-decomposition. The identified lumpable states are considered as a single state in the reduced model, and are chosen such that we may assume that they reach QSS quickly compared to the surrounding dynamics. This QSS property is the basis for the second step: to identify analytical back-translation expressions, describing the relations between the states and parameters in the reduced and the original model. These back-translation expressions are centred around the fraction parameters *η*, which represent the fraction of a lumped state to the total lump. The fraction parameters are calculated through calculations of the QSS steady-state concentrations of the lumped parameters, which are calculated analytically through the analytical inversion of the matrix .

We have developed and tested the method using two previously published models for photosynthesis. Notably, the larger of these two models was reduced from 26 to 6 states, where there is no visible difference when comparing simulations from the two models. Here it should be noted that this comparison can be done either in the space of the reduced model, or in the original state space, and that the reduced model does not have to be simulated again to obtain time-series for those detailed states that are of interest. It is because of this property that the resulting models may be viewed as two degrees of zooming of the same model; this is a conceptually important step forward in terms of model reduction, and we believe that it will prove to be very useful in modelling situations, and that it will stimulate much future research. In line with this, it should finally be said that even though most of the results and derivations in this paper are for linear models, we have shown that the same concepts and methods are applicable to nonlinear models as well, and that we are currently working on a similarly general theory for a commonly occurring class of nonlinear models.

## Authors' contributions

All four authors contributed to the development of the theory and analysis of the results. Nevertheless, HS initiated the original M.Sc. project including reduction of linear models with preserved interpretability, GC developed the concepts concerning back-translation and zooming, MS developed most of the aspects of the actual method and did most of the calculations, and MJ supervised in particular the later stages. MS, GC and MJ wrote the article. All authors read and approved the final manuscript.

## Authors' information

**Mikael Sunnåker **has a B.Sc. in Engineering Physics and a M.Sc. in Complex Adaptive Systems from Chalmers University of Technology. In January 2007 he took a position as an applied researcher at the department for Systems Biology and Bioimaging at the Fraunhofer-Chalmers Research Centre for Industrial Mathematics. Since January 2009 he is a PhD student at the department for Biosystems Science and Engineering at the Eidgenössische Technische Hochschule (ETH), and Competence Center for Systems Physiology and Metabolic Diseases, in Zurich. His research interests include methods for network identification, model reduction, and parameter estimation of biochemical reaction systems.

**Henning Schmidt **received a double M.Sc. degree in Electrical Engineering from the TU Darmstadt and SUPELEC in Paris 1997, and a Ph.D. degree in control theory from the Royal Institute of Technology in Stockholm 2004. Following his Ph.D. time he joined the Fraunhofer-Chalmers Research Centre for Industrial Mathematics where he worked on topics including modelling and analysis of biochemical reaction systems and the development of related computational tools. Since December 2008 he is working in the Modelling&Simulation group at Novartis Pharma AG in Basel.

**Mats Jirstrand **received a M.Sc. degree in applied physics and electrical engineering 1994, a Lic.Eng. degree in automatic control 1996, and a Ph.D. degree in automatic control 1998 all from Linköping University. In 2004 he was appointed associate professor in Automatic Control at the School of Electrical Engineering, Chalmers University of Technology. His research interests include modelling methodology, system identification, and model reduction, in particular for biological and medical applications. He is currently the Head of Department for Systems Biology and Bioimaging at the Fraunhofer-Chalmers Research Centre for Industrial Mathematics.

**Gunnar Cedersund **has a M.Sc. in theoretical physics. Since his master thesis he has been working with systems biology. He is interested both in methodological developments, summarized in the core-box modeling framework, and in concrete modeling of biological systems related to glucose homeostasis. His Ph.D. time was placed in two different electrical engineering groups, and he is currently co-leading the joint experimental/theoretical group "Diabetes and Integrative Systems Biology" at the Linköping university.

## Supplementary Material

Additional file 1 **Model reduction algorithm**. A Matlab function that constitutes our implementation of the model reduction algorithm (for linear models) presented in this paper. The function requires the systems biology toolbox and the symbolic math toolbox for Matlab to be installed. Click here for file

Additional file 2 **Model reduction scripts**. Matlab script that can be used to easily test the model reduction algorithm on the 6-states and 26-states models for photosynthesis that are employed in this paper. Click here for file

Additional file 3 **The 6-states model**. This is the six-states model for photosynthesis that is used in the paper. This model can be imported to Matlab in the form of an SBmodel object (requires the SBtoolbox). Click here for file

Additional file 4 **The 26-states model**. This is the 26-states model for photosynthesis that is used in the paper. The model can be imported to Matlab in the form of an SBmodel object (requires the SBtoolbox). Click here for file
